# Is intra-operative gamma probe detection really necessary for inguinal sentinel lymph node biopsy?

**DOI:** 10.1590/S1516-31802000000600003

**Published:** 2000-11-01

**Authors:** Renato Santos de Oliveira, Ivan Dunshe Abranches Oliveira Santos, Lydia Massako Ferreira, Fernando Augusto de Almeida, Milvia Maria Simões e Silvia Enokihara, Antonio Barbieri, Reinaldo Tovo

**Keywords:** Sentinel node, Lymphoscintigraphy, Gamma detection, Lymphatic mapping, Linfonodo sentinela, Linfocintilografia, detecção gama, Mapeamento linfático

## Abstract

**CONTEXT:**

Sentinel node (SN) biopsy has changed the surgical treatment of malignant melanoma. The literature has emphasized the importance of gamma probe detection (GPD) of the SN.

**OBJECTIVE:**

Our objective was to evaluate the efficacy of patent blue dye (PBD) and GPD for SN biopsy in different lymphatic basins.

**DESIGN:**

Patients with cutaneous malignant melanoma in stages I and II were submitted to biopsy of the SN, identified by PBD and GPD, as part of a research project.

**SETTING:**

Patients were seen at Hospital São Paulo by a multidisciplinary group (Plastic Surgery Tumor Branch, Nuclear Medicine and Pathology).

**PATIENTS:**

64 patients with localized malignant melanoma were studied. The median age was 46.5 years. The primary tumor was located in the neck, trunk or extremities.

**INTERVENTIONS:**

Preoperative lymphoscintigraphy, lymphatic mapping with PBD and intraoperative GPD was performed on all patients. The SN was examined by conventional and immunohistochemical staining. If the SN was not found or contained micrometastases, only complete lymphadenectomy was performed.

**MAIN MEASUREMENTS:**

The SN was identified by PBD if it was blue-stained, and by GPD if demonstrated activity five times greater than the adipose tissue of the neighborhood.

**RESULTS:**

Seventy lymphatic basins were explored. Lymphoscintigraphy showed ambiguous drainage in 7 patients. GPD identified the SN in 68 basins (97%) and PBD in 53 (76%). PBD and GPD identified SN in 100% of the inguinal basins. For the remaining basins both techniques were complementary. A metastatic SN was found in 10 basins. Three patients with negative SN had recurrence (median follow-up = 11 months).

**CONCLUSION:**

Although both GPD and PBD are useful and complementary, PBD alone identified the SN in 100% of the inguinal lymphatic basins.

## RESUMO

**CONTEXTO:**

A biópsia de linfonodo sentinela (LS) mudou a abordagem cirúrgica do melanoma maligno. A literatura tem enfatizado a importância da detecção gama intra-operatória (DG) do LS.

**OBJETIVO:**

Nosso objetivo é avaliar a eficácia do corante azul patente (AP) e da DG na biópsia de LS em diferentes bases linfáticas.

**TIPO DE ESTUDO:**

Pacientes portadores de melanoma maligno cutâneo foram submetidos à biópsia do LS, usando AP e DG como parte de um projeto de pesquisa.

**LOCAL:**

Hospital São Paulo, grupo multidisciplinar (cirurgião oncológico, médico nuclear e patologista).

**PACIENTES:**

Foram estudados 64 pacientes portadores de melanoma maligno localizado, com idade mediana de 46,5 anos. O sítio primário estava localizado no pescoço, tronco e nos membros.

**INTERVENÇÕES:**

Linfocintilografia pré-operatória, mapeamento linfático com AP e DG foram realizados em todos os pacientes. O LS foi examinado por histopatologia convencional e imunohistoquímica. Quando o LS não foi encontrado ou continha micrometástases, linfadenectomia completa da base linfática foi realizada.

**VARIÁVEIS ESTUDADAS:**

O LS foi considerado como identificado pelo AP se corado em azul e pela DG quando demonstrou pelo menos 5 vezes mais atividade do que o tecido gorduroso vizinho.

**RESULTADOS:**

Foram exploradas 70 bases linfáticas. A linfocintilografia mostrou drenagem ambígua em 7 pacientes. DG identificou o LS em 68 bases linfáticas (97%) e o AP o fez em 53 bases (76%). Os dois métodos identificaram separadamente 100% dos LS inguinais. Nas demais bases, as técnicas foram complementares. O LS estava invadido por células tumorais em 10 bases. Três pacientes com LS negativo apresentaram recorrência (seguimento mediano de 11 meses).

**CONCLUSÃO:**

Embora o emprego de AP e GP na pesquisa de LS sejam complementares, o AP demonstrou ser um método suficiente para a localização do LS inguinal.

## INTRODUCTION

Surgical treatment of regional lymph nodes in cutaneous malignant melanoma stage I and II is still controversial.^1-4^ The main problem is to characterize which patients must have lymphadenectomy. In 13.3% of stage I and 20% of stage II patients, clinically hidden lymph node metastases are removed at an early stage with elective lymph node dissection. In the remaining cases, no tumor is found in the lymph nodes and these patients receive excessive treatment and are submitted to the risk of postoperative complications.^5-11^ Balch et al. characterized a subgroup of patients under 60 years old with tumor thickness of 1-4 mm who benefited from elective lymph node dissection.^12,13^

The concept of sentinel lymph node (SN), the first node in the lymphatic basin that drains the area of the primary tumor, has changed the surgical approach in melanoma patients. This technique involves four procedures: pre-operative lymphoscintigraphy, intraoperative lymphatic mapping, intraoperative gamma-probe detection (GPD) and histopathology. Pre-operative lymphoscintigraphy identifies the lymphatic basins at risk for metastatic disease, anomalous drainage and intransit metastasis. Intraoperative lymphatic mapping with a vital dye (we have used patent blue dye, PBD) anatomically and functionally mimics the lymphatic spread of neoplastic cells from the primary site to the lymphatic basin. Blue staining of lymphatic vessels allows identification of the SN, as initially demonstrated by Morton et al.^14,15^

GPD allows better localization of SN with less extensive dissection.^16^ The SN is submitted to conventional histopathological examination (hematoxylin-eosin) and immunohistochemical staining with HMB 45 antigen and S-100 protein. Only patients with positive SN are submitted to complete lymphadenectomy.^17,18^ The accuracy of SN in predicting the histopathological status of the lymphatic basin has been confirmed by several groups around the world.^19-23^

SN biopsy has changed surgical treatment of melanoma. The literature has emphasized the importance of GPD in the SN biopsy. Before introducing GPD in our practice, we had used PBD alone with very good results for lymph nodes of the inguinal region. Our objective was to evaluate the efficacy of PBD and GPD in the SN biopsy of different lymphatic basins.

The role of PBD and GPD in the identification of the SN in different lymphatic basins of cutaneous malignant melanoma patients was studied.

## METHODS

### Setting.

Patients were treated at Hospital São Paulo of the Universidade Federal de São Paulo/Escola Paulista de Medicina (UNIFESP/EPM) by a multidisciplinary group (Plastic Surgery Tumor Branch, Nuclear Medicine and Pathology).

### Patients.

Sixty-four patients with clinically localized malignant melanoma with Breslow thickness equal to or greater than 0.8 mm were enrolled. Excision biopsy of the primary tumor was performed at most 60 days before the sentinel lymph node biopsy. Informed consent was obtained from all patients. Of the 64 patients, 31 were men and 33 women. The median age was 46.5 years old (range, 18-81 years old). The median thickness of the primary tumor was 1.3 mm (range, 0.8-7.0 mm). Thirty-eight patients were at clinical stage IB, 19 IIA and 7 IIB. The primary sites were the neck,^2^ trunk^32^ and extremities.^30^ Clinicopathologically, there were 4 accrual melanoma, 36 superficial spreading melanoma and 24 nodular melanoma. Ulceration was present in 9 cases *(Table 1).*

**Table 1:** General patient characteristics

Sex	31 Men : 33 Women
**Age**	18 - 81 years (median = 46.5 years)
**Breslow**	0.8 - 7 mm (median = 1.3 mm)
**Primary site**	
Neck	2
Trunk	32
Members	30
**Clinical Stage**	
IB	38
IIA	19
IIB	7
**Histopathological type**	
Superficial spreading	36
Nodular	24
Accrual	4
**Ulceration**	9

### Interventions.

Before operation, all patients underwent lymphoscintigraphy to characterize the lymphatic basin of drainage and to localize the SN. A dose of 250 microcurie (^99m^TC-dextran 500) was injected intradermally at 2 to 4 points around the biopsy scar or the melanoma lesion if still present.

Immediately afterwards, dynamic images were obtained to visualize the lymphatic drainage. Dynamic acquisition of images continued for a minimum of 20 min to a maximum of 45 min. Anterior and lateral static images, using a dual-head gamma camera (Eucint Helix HR), were obtained until the SN was found. The last image was obtained after 2 hours. These images were complemented by oblique views whenever the injection site could obscure the SN. The position of the SN was then marked on the skin.

In the operating room, a similar injection of ^99m^TC-dextran was given for GPD. A convenient site for incision was confirmed and determined using a Neoprobe 1500 device as a gamma probe detector (Columbus, OH). To consider a node as an SN it had to demonstrate at least 5 times more activity than the adipose tissue of the neighborhood.

Intraoperative lymphatic mapping with patent blue dye (PBD) was performed according to the technique described by Morton et al.^12^ A small incision was made, and the subcutaneous tissue was explored in the search for a blue-stained lymphatic channel. The channel was carefully dissected down to the blue-stained SN.

After the biopsy was performed, the SN (*ex vivo*) and the operative field were checked by probe. The excised SN was sent for pathological examination, using a paraffin hematoxylin-eosin section and immunohistochemical staining with S-100 protein and HMB- 45 antigen. Only when micrometastases were present was a formal regional lymph node dissection performed. The average follow-up for this group of patients was 11 months (range, 1 - 25 months).

### Main measurement.

The SN was identified by PBD if it was blue-stained and by GPD if it demonstrated at least five times more activity than the adipose tissue around it.

## RESULTS

Seventy lymphatic basins were explored. Lymphoscintigraphy showed ambiguous drainage in 7 patients. The distribution of the basins were: axillary,^35^ inguinal^29^ and cervical.^6^ In all cases PBD and GPD were performed, identifying a total of 98 SN (1.4 SN per lymphatic basin). GPD identified the SN in 68 basins (97%)and PBD in 53 basins (76%). In the axillary region, GPD identified SN in 34 basins (97%) and PBD in 22 basins (63%). In cervical basins, GPD identified SN in 5 (83%) and PBD in 2 (33%). For the 29 inguinal basins, both GPD and PBD identified 100% of the SN *(Table 2).*

**Table 2:** Percentage of SN identification by PBD and GPD techniques for lymphatic basins

Technique	Inguinal (29)	axillary (35)	Cervical (6)	Total of basins (70)	
PBD	100%	63%	33%	76%	GPD
100%	97%	83%	97%		

A tumor-positive SN was found in 10 patients (8 on permanent sections and 2 on immunohistochemistry only). Regional node dissection was performed on all 10 patients with tumor-containing SN. In addition to the SN, other involved nodes were found in three patients. Although with a short follow-up (11 months), three patients with a tumor-negative SN biopsy had a relapse: one in the same basin, another developed intransit metastasis and the third had liver metastasis (Breslow = 1.2 mm, 2.4 mm and 2.3 mm, respectively). Postoperative complications were seen in four patients, which were clinically treated (wound infection, hematoma and seroma).

## DISCUSSION

The SN procedure appears to be a reliable method for regional location microstaging of melanoma patients with clinically hidden lymph node metastases. The pathological status of SN is the most important prognostic factor for tumor recurrence.^24,25^

When using only PBD, the literature has registered a lower identification rate for the SN (85%) than with GPD (98-100%).^26-29^ Similar data were obtained in our study (PBD = 76% and GPD = 97%). Subgroup analysis of the basins showed that GPD and PBD identified 100% of the inguinal SN. For the remaining basins (axillary and cervical), GPD and PBD were complementary, but GPD identified more SN than PBD. Lymphoscintigraphy had a crucial role in localizing the SN basin and allowing skin localization of the SN, especially in the inguinal region.

Some authors have questioned whether there is any role for PBD in the SN biopsy,^27^ emphasizing the role of GPD in the outpatient clinic. Others indicate PBD as a standard procedure.^30^ Our data showed that PBD alone is a very good technique for the inguinal SN, posing a different question: is intra-operative gamma probe detection really necessary for inguinal sentinel lymph node biopsy? PBD simplifies the procedure, can be done without an intraoperative gamma detection device, and improves the cost / benefit relationship.

Three patients with tumor-negative SN biopsy had relapses. Perhaps a more powerful method for evaluating the SN status would be able to identify hidden micrometastases in those negative SN.

We are introducing detection of m-RNA thyrosinase in the SN by the reverse transcriptase polymerase chain reaction (RT-PCR). The sentinel node biopsy technique will contribute to obtaining better knowledge of the natural history of melanoma cell dissemination.
